# Performance of the nontreponemal tests and treponemal tests on cerebrospinal fluid for the diagnosis of neurosyphilis: A meta-analysis

**DOI:** 10.3389/fpubh.2023.1105847

**Published:** 2023-02-02

**Authors:** Jia-Wen Xie, Mao Wang, Ya-Wen Zheng, Yong Lin, Yun He, Li-Rong Lin

**Affiliations:** ^1^Center of Clinical Laboratory, School of Medicine, Zhongshan Hospital of Xiamen University, Xiamen University, Xiamen, China; ^2^School of Medicine, Institute of Infectious Disease, Xiamen University, Xiamen, China

**Keywords:** neurosyphilis, nontreponemal tests, treponemal tests, serological assays, diagnostic performance

## Abstract

**Background:**

Nontreponemal and treponemal tests for analyzing cerebrospinal fluid to confirm the existence of neurosyphilis have been widely used, so we aim to evaluate and compare their performance on the cerebrospinal fluid in the diagnosis of neurosyphilis.

**Methods:**

We conducted a systematic literature search on five databases and utilized a bivariate random-effects model to perform the quantitative synthesis.

**Results:**

Nontreponemal tests demonstrated a pooled sensitivity of 0.77 (95% CI: 0.68–0.83), a pooled specificity of 0.99 (95% CI: 0.97–1.00), and a summary AUC of 0.97 (95% CI: 0.95–0.98). The pooled sensitivity, pooled specificity, and summary AUC of treponemal tests were 0.95 (95% CI: 0.90–0.98), 0.85 (95% CI: 0.67–0.94), and 0.97 (95% CI: 0.95–0.98), respectively. The pooled specificity of all nontreponemal tests varied minimally (ranging from 0.97 to 0.99), with TRUST (0.83) having a higher pooled sensitivity than VDRL (0.77) and RPR (0.73). Among all treponemal tests, EIA has outstanding diagnostic performance with a pooled sensitivity of 0.99 and a pooled specificity of 0.98.

**Conclusion:**

Nontreponemal tests exhibited a higher pooled specificity, and treponemal tests exhibited a higher pooled sensitivity in diagnosing neurosyphilis on cerebrospinal fluid. TRUST may be a satisfactory substitute for VDRL. EIA is a prospective diagnostic tool that deserves further study in the future. Our study may be useful to clinical laboratories in selecting appropriate serological tests on the cerebrospinal fluid for the diagnosis of neurosyphilis.

## 1. Introduction

Syphilis, caused by *Treponema pallidum*, is a chronic bacterial infection. At any stage during the process of the illness, neurosyphilis might develop, it is a frightening complication of syphilis. Analysis of cerebrospinal fluid is often useful to confirm the existence of neurosyphilis because the disease can be asymptomatic or manifest in various ways (1). Numerous approaches for analyzing cerebrospinal fluid have been developed, with serological assays being the most commonly used diagnostic tests. There are two serologic tests for syphilis: nontreponemal and treponemal tests, the former measuring antibodies against cardiolipin and are not specific to *Treponema pallidum*, and the latter detecting specific antibodies to *Treponema pallidum* (2, 3).

Venereal disease research laboratory test (VDRL), a nontreponemal test, is often considered the standard test for confirming the diagnosis of neurosyphilis, but a light microscope is required for detection, and the reagent needs to be produced and utilized within 2 h. Rapid plasma reagin (RPR) and Toluidine red unheated serum test (TRUST), which are accessible as commercial kits, share a similar test principle as VDRL but are much easier to perform (4). Whether RPR and TRUST are promising alternatives to VDRL in the detection of cerebrospinal fluid neurosyphilis needs to be fully evaluated.

When applying treponemal tests to diagnose neurosyphilis, more and more laboratories are adopting Enzyme immunosorbent assays (EIA)-based treponemal tests instead of conventional treponemal tests, such as Fluorescent treponemal antibody absorption (FTA-ABS) or *Treponema pallidum* particle agglutination (TPPA), since it allows interfacing with the electronic medical record and achieving higher test throughput. The agreement between EIA and FTA-ABS exceeds 95% when using serum (5), however, it is not known whether the diagnostic performance of contemporary treponemal tests and conventional treponemal tests is comparable when applied to cerebrospinal fluid.

Currently, the diagnosis of neurosyphilis remains challenging, and recognizing the performance and limitations of the presently available tests is crucial. In this meta-analysis, we aim to evaluate and compare the performance of nontreponemal tests and treponemal tests on the cerebrospinal fluid in diagnosing neurosyphilis.

## 2. Materials and methods

This systematic review and meta-analysis were performed following the Preferred Reporting Items for Systematic Reviews and Meta-Analyses (PRISMA) guidelines, and the protocol of this study was registered in the PROSPERO database (CRD42022371321).

### 2.1. Search strategy and eligibility criteria

Two independent reviewers searched studies published up to 1 November 2022 in the PubMed, Web of Science, CNKI, BioRxiv, and MedRxiv databases. To find relevant papers, we employed a combination of free text and MeSH terms, the following were the main search terms: “Cerebrospinal fluid,” “Neurosyphilis,” “Sensitivity,” and “Specificity.” There are no restrictions on language. Supplementary Table S1 provides the detailed search strategy.

Papers reporting the clinical accuracy of nontreponemal tests or treponemal tests on cerebrospinal fluid for the diagnosis of neurosyphilis could be included in this meta-analysis, the sensitivity and specificity of the test should be available through article review or calculation in the full text or the Supplementary material. Reviews, editorials, letters, and case reports were excluded.

### 2.2. Data extraction

Two reviewers extracted the following data independently from all qualified papers: (1) first author and publication year; (2) the country of residence of the study participants; (3) definition of neurosyphilis or guidelines followed for the diagnosis of neurosyphilis; (4) assays evaluated in the article and true positive (TP), false positive (FP), false negative (FN), and true negative (TN) values of each assay; (5) status of the study population (HIV positive or HIV negative, symptomatic or asymptomatic), if available. Any disagreements were settled by consensus together with a third reviewer.

### 2.3. Quality assessment

The Quality Assessment of Diagnostic Accuracy Studies 2 (QUADAS-2) tool was applied to evaluate the risk of bias and applicability of each included study (6). Patient selection, index test, reference standard, and flow and timing are the four domains that the tool assesses.

### 2.4. Statistical analysis

The quantitative synthesis was performed by using a bivariate random-effects model. We utilize bivariate boxplots, qualitative *Q* tests (*p* < 0.05 indicating statistical significance), and quantitative *I*^2^ tests (ranging from 0 to 100%, with a lower value suggesting less heterogeneity) to access interstudy heterogeneity. The sensitivity analysis was used to determine if the meta-analysis's pooled effects were reliable. We calculated the pooled sensitivity, specificity, positive likelihood ratio (PLR), and negative likelihood ratio (NLR) and created forest plots to display the overall effects of all studies based on effect size and 95% confidence intervals. The summary receiver operating characteristic (SROC) curve was drawn to obtain the area under the curve (AUC). The relationship between the pre-test probability and the post-test probability is illustrated by the Probability Modifying Plot and Fagan Plot. Statistical significance was set at *P* < 0.05. All statistical analyses were conducted using STATA software (Stata Corporation, College Station, TX, USA).

## 3. Results

### 3.1. Features of eligible studies

**Figure 1:**
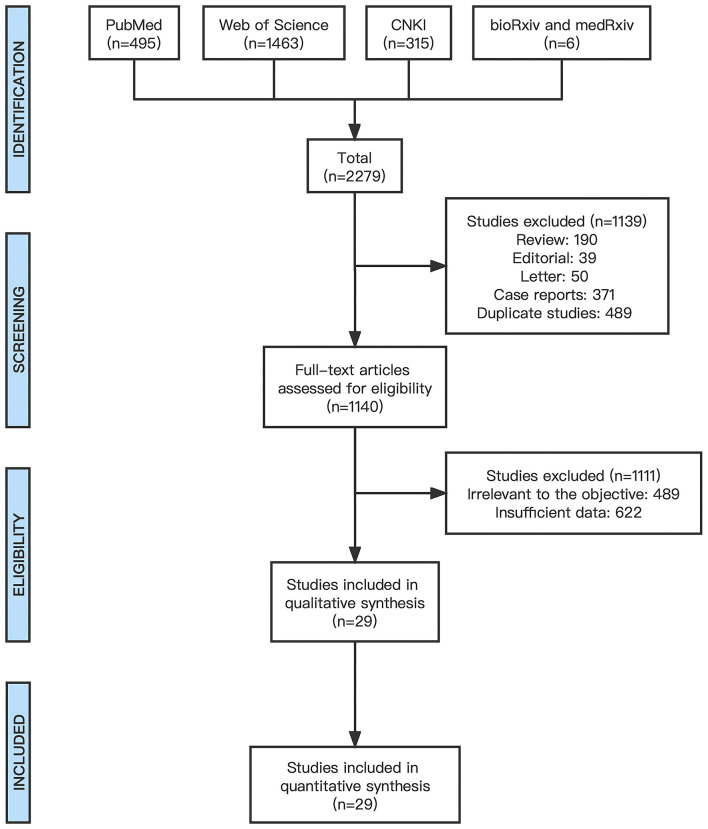
PRISMA flow diagram.

Figure 1 provided the PRISMA flow diagram, 2,279 publications were initially retrieved. The screening process resulted in the exclusion of 190 reviews, 39 editorials, 50 letters, 371 case reports, and 489 duplicate studies. A total of 1,140 full-text publications were evaluated for eligibility, 489 were deemed irrelevant to the objective, and 622 lacked sufficient data therefore they were excluded. Ultimately, our meta-analysis was limited to 29 publications (2, 4, 7–33) to perform the quantitative synthesis.

Of the included 29 articles (Table 1), 22 articles evaluated two or more serological assays. One dataset describes the clinical accuracy data of an independent serological assay, nontreponemal tests, and treponemal tests each accounting for 33 datasets, therefore generating 66 datasets (Supplementary Table S2) containing 17,733 samples. In total, data on the diagnostic accuracy of 10 serological tests were included, including three nontreponemal tests (VDRL, RPR, and TRUST) and seven treponemal tests (FTA-ABS, TPPA, *Treponema pallidum* hemagglutination assay [TPHA], Microhemagglutination Assay for *Treponema pallidum* [MHA-TP], Enzyme-linked immunosorbent assay [ELISA], EIA, and line immunoassay [INNO-LIA]). According to the provided acknowledgments, all articles were manufacturer-independent. Nearly half of the studies were conducted in China (48.3%; *n* = 14). Nine articles reported whether the cases were co-infected with HIV (4, 10, 11, 22, 26, 27, 30–32). Information on the presence or absence of symptoms in patients with neurosyphilis can be found in 13 studies (2, 4, 7, 8, 11, 13, 20–22, 27, 28, 30, 32).

**Table 1:** Characterization of included studies.

**DOI**	**References**	**Year**	**Country**	**Definition of neurosyphilis or guidelines followed for the diagnosis of neurosyphilis**	**Assays evaluated in the article**
10.1111/jdv.18604	Binnicker (3)	2022	France	Guidelines of the Centers for Disease Control (CDC) in the US	VDRL
10.1097/OLQ.0000000000001450	Marra (32)	2021	USA	Asymptomatic neurosyphilis was defined as no neurological symptoms and with CSF white blood cells (WBCs) >10/ul; Symptomatic neurosyphilis as neurological symptoms, including new vision loss and hearing loss, and CSF WBCs >10/ul	VDRL, TPPA
10.1097/OLQ.0000000000001308	Gonzalez et al. (33)	2021	USA	Reactive CSF-VDRL with or without CSF pleocytosis and their CSF abnormalities normalized after CDC-recommended neurosyphilis therapy	FTA-ABS
10.15932/j.0253-9713.2020.06.006	Li et al. (31)	2020	China	(1) Positive serum TPPA and RPR; (2) CSF-WBC >10 × 10 6/L and/or CSF-protein >500 mg/L; (3) Positive CSF FTA-ABS IgM and/or positive CSF RPR	RPR, TPPA, FTA-ABS
10.1186/s12879-019-4582-2	Lu et al. (26)	2019	China	Guidelines of the Centers for Disease Control in Europe and America	TPPA
10.19435/j.1672-1721.2018.28.002	Su et al. (24)	2018	China	Diagnostic criteria for neurosyphilis in China	ELISA, RPR, VDRL
10.1309/AJCPWSL3G8RXMCQR	Guarner et al. (18)	2016	USA	Two or more reactive/positive treponemal, nontreponemal, or PCR tests in CSF were considered neurosyphilis	VDRL, EIA, TPPA, INNO-LIA
10.1093/cid/ciw499	Vanhaecke et al. (28)	2016	France	(1) positive treponemal and nontreponemal serum test results; (2) positive CSF VDRL or positive CSF FTA-Abs test result and 1 CSF abnormality in laboratory tests, such as pleocytosis (cell count, >20/μL) or high protein levels (>0.5 g/L); and (3) clinical symptoms	VDRL
10.1016/j.cca.2016.10.018	Lin et al. (17)	2016	China	European guidelines and the US Centers for Disease Control guidelines	RPR, TPPA
10.1038/srep33569	Wang et al. (21)	2016	China	A reactive CSF-VDRL and a reactive CSF-TPPA in the absence of substantial contamination of CSF with blood	VDRL
10.1590/0004-282X20160016	Salamano et al. (30)	2015	Uruguay	VDRL+ in CSF and/or showed TPHA in CSF ≥ 320, and/or showed TPHA-albumin index ≥ 70	VDRL, TPHA
10.1186/s40001-015-0175-8	Merins and Hahn (12)	2015	Germany	Neurosyphilis was probable if two of the first three following conditions were fulfilled and in addition to that the 4th condition always had to be fulfilled: (1) Chronically progressive course of neurologic-psychiatric symptoms with phases of aggravation and partly remission. (2) Pathological CSF with mixed cellular or mononuclear pleocytosis (>4 cells/μl), blood-CSF barrier disturbance (CSF-protein >0.5 g/l or albumin quotient >7.8) and/or IgG-dominant immune response in central nervous system. (3) Beneficial effect of antibiotics on the course of the disease and/or pathological CSF (particularly pleocytosis and barrier disturbance). (4) Positive TPHA (or TPPA) and FTA-abs in serum	RPR, FTA-ABS
10.15932/j.0253-9713.2015.12.006	Li et al. (10)	2015	China	(1) neurological symptoms and signs; (2) positive serological tests; (3) abnormal cerebrospinal fluid cell counts or proteins, positive cerebrospinal fluid FTA-ABS and/or positive VDRL tests	ELISA, TPPA, RPR
10.1177/0956462413515452	Chan et al. (9)	2014	China	IUSTI 2008 guidelines	EIA
10.1128/JCM.02522-13	Zhu et al. (4)	2014	China	Symptomatic neurosyphilis was defined as the combination of clinical symptoms or signs consistent with neurosyphilis without other known causes of the clinical abnormalities, with a positive CSF-TPPA in the absence of contamination with blood; Asymptomatic neurosyphilis was defined as the combination of elevated CSF WBC count (≥10/μl) without other known causes, with a positive CSF-TPPA in the absence of contamination with blood	VDRL, RPR, TRUST
10.13381/j.cnki.cjm.201412013	Su et al. (8)	2014	China	Diagnostic criteria for neurosyphilis in China	ELISA, TRUST, VDRL
10.1159/000347109	Zhang et al. (25)	2013	China	Guidelines of the Centers for Disease Control (CDC) in the US	RPR, TPPA
10.1128/JCM.01989-13	Dumaresq et al. (27)	2013	Canada	A reactive CSF-VDRL test result and/or a CSF white blood cell (WBC) count of >20 cells/μl	FTA-ABS, TPPA, INNO-LIA
10.3760/cma.j.issn.0412-4030.2011.02.019	Lin et al. (23)	2011	China	Reactive CSF-VDRL	RPR, TPPA, FTA-ABS
10.1097/OLQ.0b013e3181f42093	Jiang et al. (11)	2011	China	CSF-VDRL positivity, or a CSF-WBC count of >5 cells/μL with CSF-Treponema pallidum particle agglutination (TPPA) positivity	VDRL, TPPA, TRUST
10.1097/OLQ.0b013e3181d877a1	Marra et al. (22)	2010	USA	Symptomatic neurosyphilis was defined as hearing or visual loss regardless of CSF abnormalities; Asymptomatic neurosyphilis was defined as a reactive CSF-VDRL or CSF WBCs > 20/μL in the absence of hearing or vision loss	VDRL
10.3760/cmad.issn.1671−8925.2010.08.023	Zheng et al. (19)	2010	China	Guidelines of the Centers for Disease Control (CDC) in the US in 1996	TPPA, TRUST
10.16252/j.cnki.issn1004-0501-2010.11.069	Hong and Rao (14)	2010	China	The Law of the People's Republic of China on the Prevention and Control of Infectious Diseases and the Measures for the Implementation of the Law of the People's Republic of China on the Prevention and Control of Infectious Diseases and the 2006 CDC Guidelines for the Prevention and Control of Sexually Transmitted Diseases	RPR, VDRL
10.1002/jcla.20254	Castro et al. (15)	2008	Portugal	All CSF samples from individuals with reactive serological tests for syphilis in sera (RPRZ1:8 and MHA-TPZ1:80) and with a reactive CSF-Fluorescent Treponemal Antibody Absorption Assay (CSF-FTA-Abs), increased proteins, and cell count	VDRL, RPR
10.1097/01.olq.0000233738.23278.4e	Paraskevas et al. (29)	2007	Greece	European guidelines	VDRL
10.1002/jcla.20147	Castro et al. (7)	2006	Portugal	Positive MHA-TP and/or FTA-Abs tests, increased number of mononuclear cells >10/mm^3^ (white blood cells [WBC]), plus reactive VDRL test in the CSF	VDRL, MHA-TP, TPPA, FTA-ABS
10.2340/00015555-0092	Woehrl and Geusau (16)	2006	Austria	Either on a reactive CSF VDRL test according to the CDC guidelines, and/or if the patient met additional criteria defined in the European STD guidelines positive CSF TPHA and/or FTA-Abs test, augmented CSF white blood cell count (>10/mm^3^), intrathecal synthesis of IgG and/or IgM quantified by the respective indices {IgG index ≥0.7; IgM index ≥ 0.10; IgG index = [[total CSF IgG (mg/l)] × [serum albumin (mg/l)]/[total serum IgG (mg/l)] × [CSF albumin (mg/l)]]; IgM index likewise}	VDRL, FTA-ABS, THPA
10.1128/JCM.24.5.736-740.1986	Lee et al. (20)	1986	USA	Reactive VDRL or FTA-ABS test in CSF	ELISA, VDRL, MHA-TP, FTA-ABS
10.1111/j.1365-4362.1983.tb02129.x	Lee et al. (13)	1983	USA	(1) positive CSF FTA-ABS and neurologic findings suggestive of neurosyphilis; (2) positive blood FTA-ABS in patients with typical neurologic findings of neurosyphilis	VDRL, FTA-ABS

### 3.2. Quality assessment

Based on the QUADAS-2 tool, Supplementary Table S3 presents the quality of the papers included in our meta-analysis. Regarding the patient selection domain, 7 (24.1%) publications were considered at high risk of bias as patients were not selected randomly, and case-control designs were performed. Twenty (68.9%) papers provided detailed explanations of the index test and were thus resulting in a low risk of bias. All studies were judged to have a low risk of bias in the reference standard domain. Given that all selected patients were enrolled in the analysis and received the same reference standard, 89.6% (26/29) of the articles were deemed to have a low risk of bias in flow and timing domains. In terms of applicability concerns, all domains were thought to meet the objectives of this meta-analysis.

### 3.3. Heterogeneity analysis

According to the statistical analysis of Stata, the proportion of heterogeneity likely due to the threshold effect for nontreponemal tests and treponemal tests was 0.08 and 0.15, respectively, suggesting that the heterogeneity of this meta-analysis was unrelated to the threshold effect. *I*^2^ for sensitivity and specificity in nontreponemal tests was 93.95 and 94.36, and in treponemal tests was 97.45 and 98.87. Furthermore, as shown in Supplementary Figure S1, the bivariate boxplot of nontreponemal tests and treponemal tests demonstrated three and five outliers, respectively, indicating the existence of interstudy heterogeneity. Therefore, the traditional fixed effect model was inapplicable to our analysis, hence the bivariate random-effects model was employed in the following quantitative synthesis.

### 3.4. Sensitivity analysis

For both nontreponemal tests and treponemal tests, it can be seen visually from their Goodness-Of-Fit (Supplementary Figures S2A, E) and Bivariate Normality analyses (Supplementary Figures S2B, F) to conclude that the statistical findings we obtained were relatively robust because the data were primarily focused on the diagonal. In nontreponemal tests, influence analysis (Supplementary Figure S2C) identified three influential observations, while outlier detection (Supplementary Figure S2D) depicted no outlier studies. When removing the three datasets, only the pooled specificity decreased minimally in the overall effect (Sensitivity: 0.77 vs. 0.77; Specificity: 0.99 vs. 0.98; AUC: 0.97 vs. 0.97). In treponemal tests, influence analysis (Supplementary Figure S2G) and outlier detection (Supplementary Figure S2H) demonstrated two and one influencing points, respectively, where the influencing point of outlier detection overlaps with one of that in influence analysis. The overall pooled effects dropped slightly after the two datasets were excluded (Sensitivity: 0.95 vs. 0.95; Specificity: 0.85 vs. 0.82; AUC: 0.97 vs. 0.96). The results indicated that these outliers did not influence our findings.

### 3.5. Diagnostic performance

**Figure 2:**
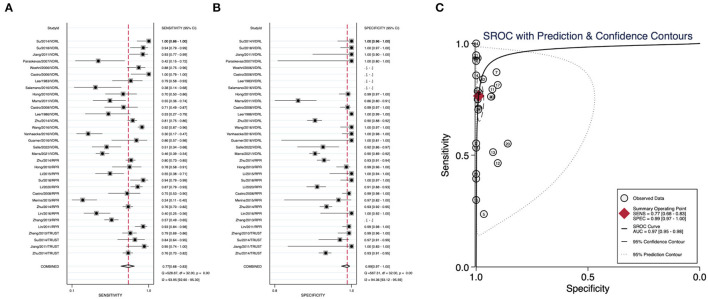
Pooled sensitivity, pooled specificity, and summary ROC curve of nontreponemal tests. **(A)** Forest plots of pooled sensitivity. **(B)** Forest plots of pooled specificity. **(C)** Summary ROC curve and its area under the curve.

**Figure 3:**
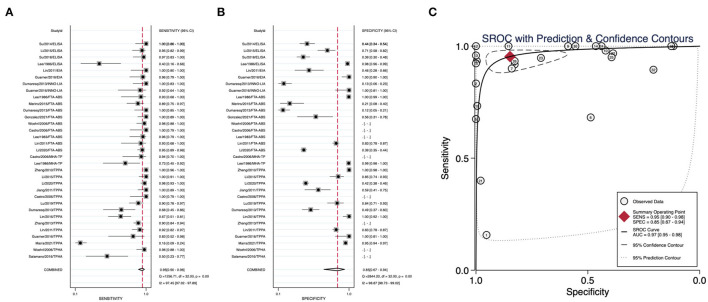
Pooled sensitivity, pooled specificity, and summary ROC curve of treponemal tests. **(A)** Forest plots of pooled sensitivity. **(B)** Forest plots of pooled specificity. **(C)** Summary ROC curve and its area under the curve.

We summarized the datasets of independent assays belonging to the nontreponemal tests or treponemal tests separately to calculate their corresponding diagnostic performance parameters. Nontreponemal tests demonstrated a pooled sensitivity of 0.77 (95% CI: 0.68–0.83), a pooled specificity of 0.99 (95% CI: 0.97–1.00) and a summary AUC of 0.97 (95% CI: 0.95–0.98) (Figure 2). The pooled sensitivity, pooled specificity, and summary AUC of treponemal tests were 0.95 (95% CI: 0.90–0.98), 0.85 (95% CI: 0.67–0.94), and 0.97 (95% CI: 0.95–0.98), respectively (Figure 3).

**Figure 4:**
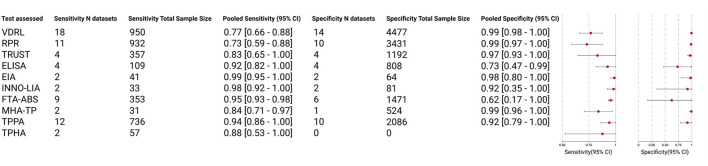
Clinical accuracy of 3 nontreponemal tests and 7 treponemal tests.

As shown in Figure 4, the pooled sensitivity of all treponemal tests, including ELISA, EIA, INNO-LIA, FTA-ABS, MHA-TP, TPPA, and TPHA (ranging from 0.84 to 0.99) is higher than that of all nontreponemal tests, including VDRL, RPR, and TRUST (ranging from 0.73 to 0.83). EIA obtained the highest pooled sensitivity of 0.99, followed by INNO-LIA with a pooled sensitivity of 0.98. The pooled specificity of the three nontreponemal tests ranged from 0.97 to 0.99. No relevant data were available for TPHA to calculate the pooled specificity, therefore six of the seven treponemal tests yielded data for pooled specificity with a range from 0.62 to 0.99, the pooled specificity for VDRL, RPR, and MHA-TP all achieved 0.99, followed by EIA (0.98) and TRUST (0.97). Among these 10 serological assays, only EIA had pooled sensitivity (0.99) and pooled specificity (0.98) both exceeding 0.95.

**Figure 5:**
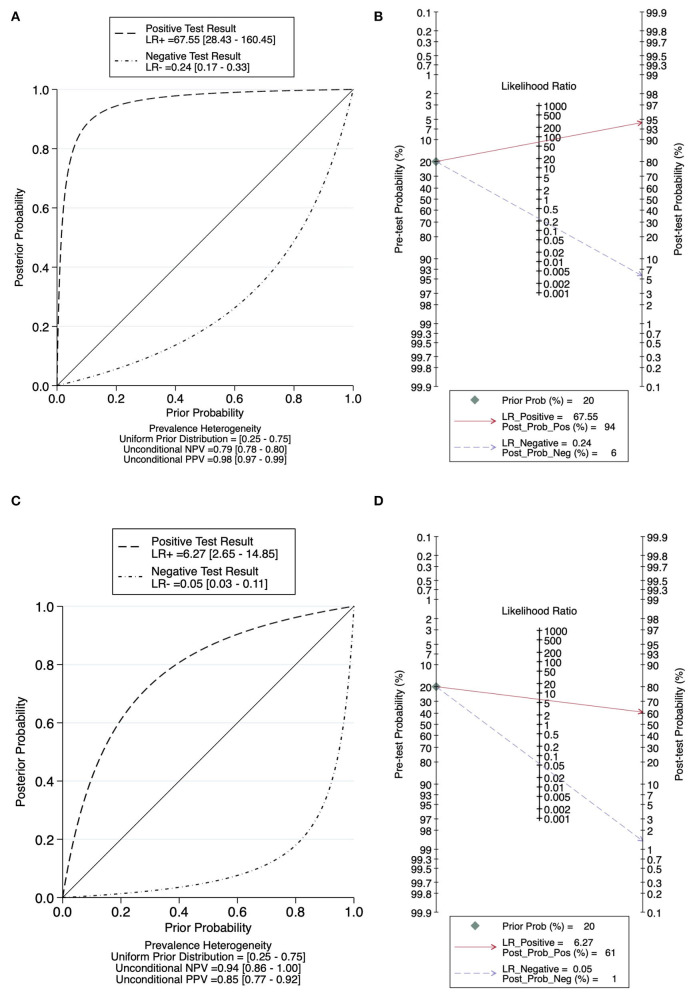
Probability Modifying Plot and Fagan Plot for evaluating the diagnostic value. **(A)** Probability Modifying Plot of nontreponemal tests. **(B)** Fagan Plot of nontreponemal tests. **(C)** Probability Modifying Plot of treponemal tests. **(D)** Fagan Plot of treponemal tests.

Figure 5 plotted the relationship between pre-test and *post-test* probability based on positive likelihood ratios and negative likelihood ratios. In contrast to tests with more informative negative results, which create curves tending toward the (1, 0) location, tests with more informative positive results have curves that tend toward the (0, 1) location. Nontreponemal tests yield a pooled positive likelihood ratio of 67.55 (95% CI: 28.43–160.45) and a pooled negative likelihood ratio of 0.24 (95% CI: 0.17–0.33) (Figure 5A), and treponemal tests generated a pooled positive likelihood ratio of 6.27 (95% CI: 2.65–14.85) and a pooled negative likelihood ratio of 0.05 (95% CI: 0.03–0.11) (Figure 5B). Based on the statistical analysis of all the data we included, the prevalence of neurosyphilis in this meta-analysis, which is the pretest probability, was 20.00%. According to the Fagan Plot in Figures 5B, D, the positive *post-test* probabilities of nontreponemal tests and treponemal tests were 94.00 and 61.00%, respectively, and the negative *post-test* probabilities were 6.00 and 1.00%, respectively. In comparison, nontreponemal tests produced more informative positive results, while treponemal tests produced more informative negative results.

### 3.6. Subgroup analysis

We subdivided the included datasets and performed subgroup analysis according to the following four characteristics: asymptomatic, symptomatic, HIV-negative, and HIV-positive (Table 2). In all subgroups, when we compared nontreponemal tests with treponemal tests, nontreponemal tests always had a higher pooled specificity, whereas treponemal tests always had a higher pooled sensitivity, regardless of the asymptomatic or symptomatic subgroup, HIV-negative or HIV-positive subgroup.

**Table 2:** Pooled sensitivity and specificity among subgroups of studies.

**Subgroups**	**Sensitivity N datasets**	**Sensitivity total sample size**	**Pooled sensitivity (95% CI)**	**Specificity N datasets**	**Specificity total sample size**	**Pooled specificity (95% CI)**
**Asymptomatic**						
Nontreponemal tests	8	397	0.63 [0.54–0.71]	1	813	0.92 [0.91–0.92]
Treponemal tests	6	118	0.90 [0.07–1.00]	2	437	0.67 [0.14–0.96]
**Symptomatic**						
Nontreponemal tests	11	755	0.86 [0.75–0.92]	8	1,185	0.89 [0.88–0.90]
Treponemal tests	7	110	0.99 [0.49–1.00]	6	486	0.58 [0.23–0.87]
**HIV-negative**						
Nontreponemal tests	10	1,073	0.77 [0.64–0.85]	9	4,621	0.92 [0.90–0.94]
Treponemal tests	6	356	0.95 [0.71–0.99]	6	1,363	0.72 [0.49–0.87]
**HIV-positive**						
Nontreponemal tests	2	134	0.47 [0.41–0.53]	2	1,676	0.91 [0.90–0.92]
Treponemal tests	3	120	0.69 [0.29–0.93]	3	820	0.58 [0.20–0.89]

When comparing the asymptomatic and symptomatic subgroups, the same situation occurred for nontreponemal tests and treponemal tests, whose pooled sensitivity in the asymptomatic subgroup was lower than that in the symptomatic subgroup (nontreponemal tests: 0.63 vs. 0.86, treponemal tests: 0.90 vs. 0.99), while the pooled specificity was higher in the asymptomatic subgroup than in the symptomatic subgroup (nontreponemal tests: 0.92 vs. 0.89, treponemal tests: 0.67 vs. 0.58).

In the HIV-negative subgroup, the pooled sensitivity of both nontreponemal tests and treponemal tests was higher than that of the HIV-positive subgroup (nontreponemal tests: 0.77 vs. 0.47, treponemal tests: 0.92 vs. 0.91), and the pooled specificity was also higher than that of the HIV-positive subgroup (nontreponemal tests: 0.95 vs. 0.69, treponemal tests: 0.72 vs. 0.58).

## 4. Discussion

It is the first meta-analysis to summarize data from all relevant publications to evaluate the diagnostic accuracy of nontreponemal and treponemal tests in diagnosing neurosyphilis. We found that nearly half of the studies were conducted in China, we speculate that this may be attributed to the Chinese government's emphasis on the prevention and control of neurosyphilis, which then led to an increase in independent validations of neurosyphilis testing in China. In recent years, the incidence of neurosyphilis has been increasing in China (34, 35). A 10-year syphilis control plan was launched by the Chinese government in 2010, meanwhile, due to the irreversibility of neurosyphilis, the Chinese Center for Disease Control has set up a neurosyphilis-specific consortium and established sentinel surveillance for neurosyphilis nationwide (36). To present, no VDRL kits in China have received SFDA (State Food and Drug Administration) approval, and the “China National Guidelines for the Diagnosis and Treatment of Syphilis, Gonorrhea and Chlamydia Trachomatis Infection (2020)” suggested the RPR and TRUST for substitute tests (34). The results of the CSF-FTA-ABS test can be utilized as a diagnostic indicator of neurosyphilis, in accordance with the guidelines for the diagnosis and treatment of syphilis in China, and in the absence of these conditions, the CSF-FTA-ABS test could be substituted with the CSF-TPPA test (37).

The pooled sensitivities of nontreponemal tests and treponemal tests were 0.77 (95% CI: 0.68–0.83) and 0.95 (95% CI: 0.90–0.98), respectively, and the pooled specificities were 0.99 (95% CI: 0.97–1.00) and 0.85 (95% CI: 0.67–0.94), respectively. Comparatively, nontreponemal tests exhibited a higher pooled specificity, and treponemal tests exhibited a higher pooled sensitivity, this conclusion also stood in the subgroup analysis. The diagnosis of a disease can be confirmed by a positive likelihood ratio >10, whereas a negative likelihood ratio <0.1 eliminates the probability of disease (38). Nontreponemal tests and treponemal tests demonstrated a pooled positive likelihood ratio of 67.55 (95% CI: 28.43–160.45) and 6.27 (95% CI: 2.65–14.85) and a pooled negative likelihood ratio of 0.24 (95% CI: 0.17–0.33) and 0.05 (95% CI: 0.03–0.11), respectively. In the diagnosis of neurosyphilis, nontreponemal tests exhibited a higher pooled specificity and produced more informative positive results, while treponemal tests exhibited a higher pooled sensitivity and produced more informative negative results. According to the Guideline for performance characteristics of immunological qualitative tests (39), the sensitivity of the screening test should be higher than 95%, and the specificity of the confirmatory test should be higher than 98%. We speculated that maybe treponemal tests were more suitable for screening tests, and nontreponemal tests might be better suited for confirmation tests.

For diagnostic purposes, Gonzalez et al. found that comparing the false-negative results of both the Treponemal test and the Nontreponemal test with the true-positive results, the false-negative results had lower CSF-VDRL titers and fewer cerebrospinal fluid white blood cells, which may be related to cerebrospinal fluid dilution (33).

Among the three nontreponemal tests, TRUST had the highest pooled sensitivity (0.83), although the pooled specificity (0.97) was slightly lower than that of VDRL (0.99) and RPR (0.99). TRUST is cost-saving, simple to manufacture, commercially available to be used in common hospitals, and could be carried out quantitatively to help with the follow-up of treatment plans (4, 11). VDRL has long been regarded as the best nontreponemal test for the diagnosing of neurosyphilis, our findings suggest that TRUST may be a satisfactory substitute for VDRL.

According to the antigens used in the assays, the treponemal tests can be divided into three categories: whole protein (FTA-ABS), soluble protein (MHA-TP, TPPA, and TPHA), and recombinant protein (ELISA, EIA, and INNO-LIA). The highest pooled sensitivity in each type was FTA-ABS (0.84), TPPA (0.94), and EIA (0.99), respectively. Park et al. evaluated the sensitivity and specificity of treponemal tests for the diagnosis of syphilis and suggested that more data on the comparative performance of FTA-ABS, TPPA, and chemiluminescence immunoassay (CIA) for diagnosing neurosyphilis on the cerebrospinal fluid are needed in the future (40), and we discovered that EIA had the highest pooled sensitivity and pooled specificity of these three tests. Compared to FTA-ABS and TPPA, which are manual tests, automated EIA reduces the burden on healthcare workers, furthermore, it requires less specimen volume and quicker turnaround time (18), which may meet the growing need for neurosyphilis screening using cerebrospinal fluid.

Among all serological assays, only EIA, with a pooled sensitivity of 0.99 and a pooled specificity of 0.98, achieved the required sensitivity of 0.95 and specificity of 0.95 for diagnostic tests (39). EIA may be a promising serological test for the diagnosis of neurosyphilis, and more studies are needed in the future to further evaluate the diagnostic performance of EIA for diagnosing neurosyphilis on cerebrospinal fluid.

In the symptomatic subgroup, whether employing the nontreponemal tests or the treponemal tests for the diagnosis of neurosyphilis, the pooled sensitivity was higher than that of the asymptomatic subgroup, however, the pooled specificity was lower than that of the asymptomatic subgroup. Previous studies have demonstrated that patients with symptomatic neurosyphilis are more likely to have a serologic reaction to cerebrospinal fluid syphilis testing than patients with asymptomatic neurosyphilis (41, 42), and our study further confirms this finding. This is probably attributable to the fact that abnormal cerebrospinal fluid white blood cell counts and cerebrospinal fluid protein concentrations are more common in patients with symptomatic neurosyphilis, suggesting that symptomatic neurosyphilis is related to a more severe blood-brain barrier damage and a larger inflammatory response (43). Clinical manifestations of symptomatic neurosyphilis vary and closely resemble the signs and symptoms of other neurological diseases. It is challenging to differentiate symptomatic neurosyphilis from other central nervous system disorders (44). Our study also revealed that the pooled specificity of symptomatic neurosyphilis is much lower, which means that patients with other neurological disorders may be misdiagnosed as neurosyphilis.

In the HIV-positive cohort, the pooled sensitivity and pooled specificity of nontreponemal tests were lower than in the HIV-negative cohort, and treponemal tests displayed the same results. Neurosyphilis co-infection with HIV has been proven to increase HIV viral load in cerebrospinal fluid, *Treponema pallidum* may interact with HIV since the two pathogens share the same antigen-presenting cells (45). So we suspect that HIV may have impaired antibody responses to the antigen used in nontreponemal tests and treponemal tests on CSF, however, this speculation requires subsequent experiments to verify.

There were some limitations existed in our article. CIA has been shown to be highly discriminative in the diagnosis of syphilis (46), but we did not evaluate the diagnostic performance of CIA in neurosyphilis because no literature was found on the use of CIA on cerebrospinal fluid for the diagnosis of neurosyphilis. Although we performed the subgroup analysis of symptomatic and asymptomatic neurosyphilis, we were unable to perform further subgroup analysis of neurosyphilis with different symptoms because data on the sensitivity and/or specificity of specific symptoms of neurosyphilis were not available. Meanwhile, we detected a high level of heterogeneity since there is no gold standard for the diagnosis of neurosyphilis, most of the articles we included had different definitions of neurosyphilis, which was likely to be the source of heterogeneity in our analysis. In the meantime, we initially identified 2,279 articles, but many were excluded for reasons such as not matching the topic or not providing data of sensitivity and specificity, so we ended up including only 29 articles, which could cause some degree of bias. However, the bivariate random-effects model we employed provided a relatively stable result, and the sensitivity analysis proved that our results were robust to a certain extent. Our work could serve as a starting point for future multicenter studies to help better understand how well cerebrospinal fluid serological testing performs in diagnosing neurosyphilis.

## 5. Conclusion

Nontreponemal tests yielded higher specificity than treponemal tests and might be better suited for confirmation tests, while treponemal tests were of higher sensitivity than nontreponemal tests and might be more suitable for screening tests. TRUST may be a satisfactory substitute for VDRL. EIA is a potential diagnostic tool for neurosyphilis that deserves further study in the future. Our study may be useful to clinical laboratories in selecting appropriate serological tests on the cerebrospinal fluid for the diagnosis of neurosyphilis in patients.

## Data availability statement

The original contributions presented in the study are included in the article/Supplementary material, further inquiries can be directed to the corresponding author.

## Author contributions

J-WX: methodology and writing–original draft. YH: software and investigation. Y-WZ: formal analysis and investigation. MW: validation and data curation. YL: visualization and supervision. L-RL: conceptualization and writing–review and editing. All authors contributed to the article and approved the submitted version.

## Supplementary material

Supplementary Figure S1 Bivariate boxplot for evaluating heterogeneity. **(A)** Nontreponemal tests. **(B)** Treponemal tests.

Supplementary Figure S2 Graphs for sensitivity analyses. **(A)** Goodness-Of-Fit of nontreponemal tests. **(B)** Bivariate normality of nontreponemal tests. **(C)** Influence analysis of nontreponemal tests. **(D)** Outlier detection of nontreponemal tests. **(E)** Goodness-Of-Fit of treponemal tests. **(F)** Bivariate normality of treponemal tests. **(G)** Influence analysis of treponemal tests. **(H)** Outlier detection of treponemal tests.

Supplementary Table S1 Literature search strategy.

Supplementary Table S2 Clinical accuracy data of nontreponemal and treponemal tests.

Supplementary Table S3 Quality of studies.
