# Supplementary figures and images for: Performance of the nontreponemal tests and treponemal tests on cerebrospinal fluid for the diagnosis of neurosyphilis: A meta-analysis
Source: Front Public Health. 2023 Feb 2;11:1105847. doi: 10.3389/fpubh.2023.1105847 (PMC9932918; doi:10.3389/fpubh.2023.1105847)

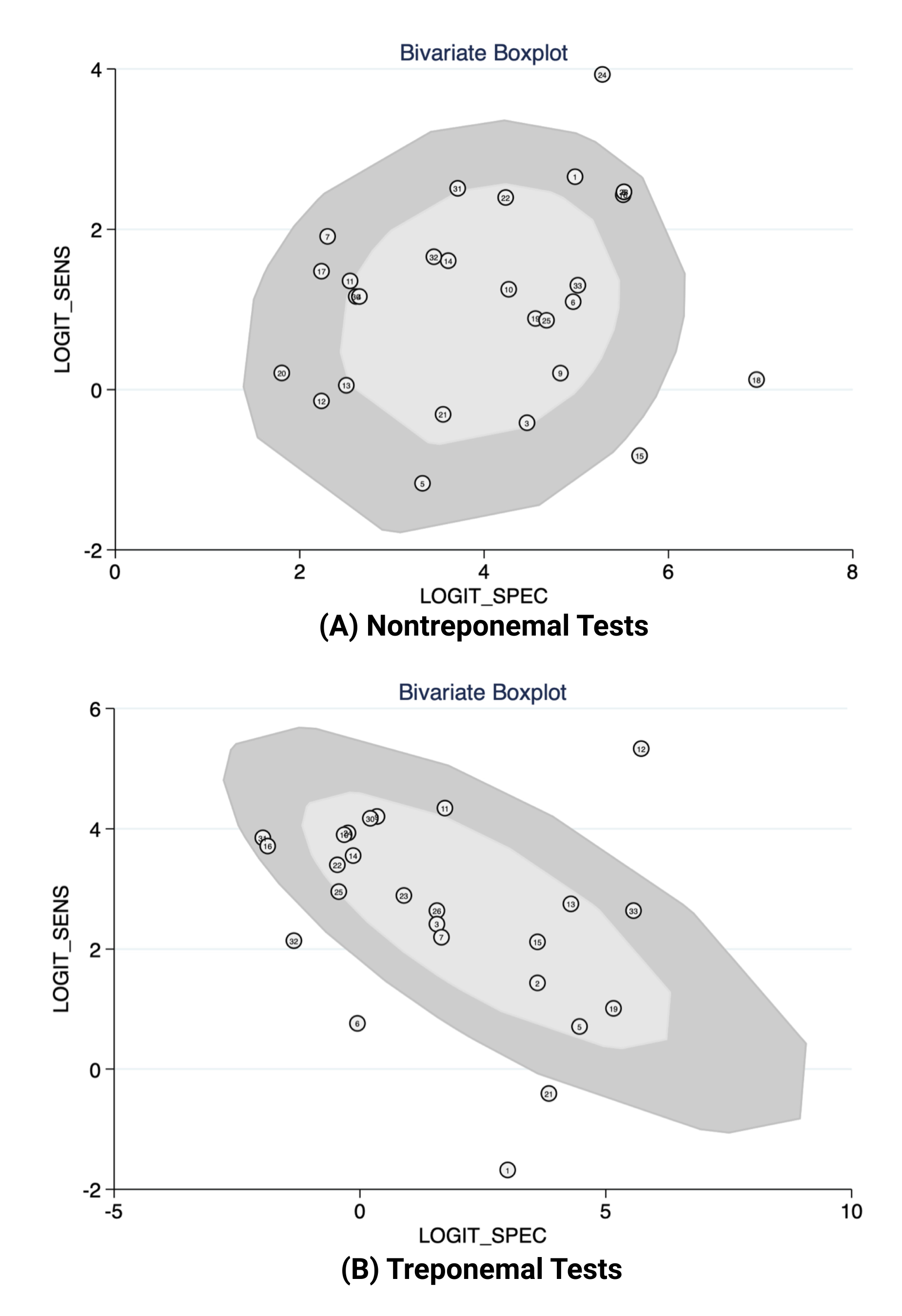

Supplement: Supplementary Figure S1 — Bivariate boxplot for evaluating heterogeneity. (A) Nontreponemal tests. (B) Treponemal tests. [file Image_1.TIFF]

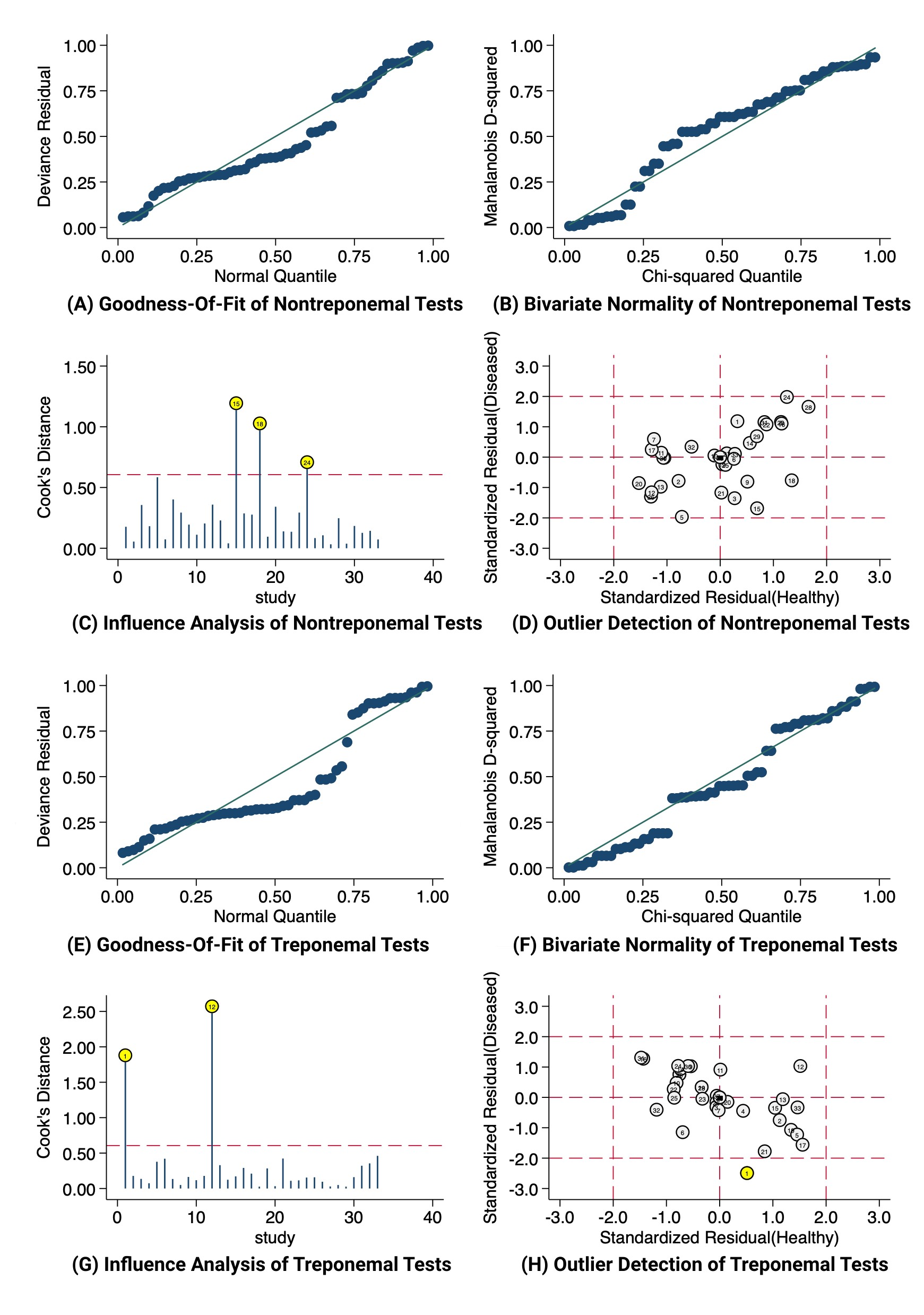

Supplement: Supplementary Figure S2 — Graphs for sensitivity analyses. (A) Goodness-Of-Fit of nontreponemal tests. (B) Bivariate normality of nontreponemal tests. (C) Influence analysis of nontreponemal tests. (D) Outlier detection of nontreponemal tests. (E) Goodness-Of-Fit of treponemal tests. (F) Bivariate normality of treponemal tests. (G) Influence analysis of treponemal tests. (H) Outlier detection of treponemal tests. [file Image_2.TIFF]
