# Supplementary material for: Performance of the nontreponemal tests and treponemal tests on cerebrospinal fluid for the diagnosis of neurosyphilis: A meta-analysis
Source: Front Public Health. 2023 Feb 2;11:1105847. doi: 10.3389/fpubh.2023.1105847 (PMC9932918; doi:10.3389/fpubh.2023.1105847)
Supplement: Supplementary Table S1 — Literature search strategy. [file Table_1.DOCX]

**Table S1**

Literature Search Strategy

| PubMed  (n=495) | (((cerebrospinal fluid[Title/Abstract]) OR (CSF[Title/Abstract])) AND ((neurosyphilis[Title/Abstract]) OR (NS[Title/Abstract]))) AND (((((sensitivity[Title/Abstract]) OR (sensitive[Title/Abstract])) OR (specificity[Title/Abstract])) OR (specific[Title/Abstract])) OR (diagnosis[Title/Abstract])) |
| --- | --- |
| Web of Science  (n=1463) | **#1 (TS=(cerebrospinal fluid)) OR TS=(CSF)**  **#2 (TS=(neurosyphilis)) OR TS=(NS)**  **#3 ((((TS=(sensitivity)) OR TS=(sensitive)) OR TS=(specificity)) OR TS=(specific)) OR TS=(diagnosis)**  **#1 AND #2 AND #3** |
| CNKI  (n=315) | SU=' **cerebrospinal fluid** ' AND SU=' **neurosyphilis** ' AND SU=' **sensitivity** '+' **specificity** '+' **sensitive** '+' **specific** '+' **diagnosis** ' |
| BioRxiv and MedRxiv  (n=6) | **"cerebrospinal fluid" and "neurosyphilis" and ("sensitivity" or "specificity" or "sensitive" or "specific" or "diagnosis")** |
