# Supplementary material for: Performance of the nontreponemal tests and treponemal tests on cerebrospinal fluid for the diagnosis of neurosyphilis: A meta-analysis
Source: Front Public Health. 2023 Feb 2;11:1105847. doi: 10.3389/fpubh.2023.1105847 (PMC9932918; doi:10.3389/fpubh.2023.1105847)
Supplement: Supplementary Table S2 — Clinical accuracy data of nontreponemal and treponemal tests. [file Table_2.DOCX]

**Table S2**

Clinical accuracy data of Nontreponemal and Treponemal Tests

| Author | TP | FN | TN | FP | SS |
| --- | --- | --- | --- | --- | --- |
| Nontreponemal Tests | | | | | |
| VDRL | | | | | |
| Marra (32) | 79 | 91 | 1525 | 163 | 1858 |
| Salle (3) | 19 | 18 | 98 | 8 | 143 |
| Guarner (17) | 12 | 2 | 18 | 0 | 32 |
| Vanhaecke (28) | 12 | 28 | 147 | 0 | 187 |
| Wang (20) | 176 | 15 | 123 | 0 | 314 |
| Zhu (23) | 171 | 39 | 833 | 89 | 1132 |
| Lee (19) | 8 | 7 | 524 | 0 | 539 |
| Castro (14) | 17 | 7 | 286 | 3 | 313 |
| Marra (21) | 16 | 13 | 146 | 24 | 199 |
| Paraskevas (29) | 5 | 7 | 17 | 0 | 29 |
| Jiang (10) | 27 | 2 | 34 | 0 | 63 |
| Su (24) | 29 | 2 | 124 | 0 | 155 |
| Su (7) | 25 | 0 | 98 | 0 | 123 |
| Hong (13) | 19 | 8 | 215 | 2 | 244 |
| Salamano (30) | 5 | 8 |  |  | 13 |
| Lee (12) | 19 | 5 |  |  | 24 |
| Castro (4) | 16 | 0 |  |  | 16 |
| Woehrl (15) | 38 | 5 |  |  | 43 |
| RPR | | | | | |
| Lin (22) | 57 | 4 | 293 | 2 | 356 |
| Zhang (25) | 85 | 64 |  |  | 149 |
| Lin (16) | 17 | 26 | 43 | 0 | 86 |
| Zhu (23) | 160 | 50 | 861 | 61 | 1132 |
| Merins (11) | 9 | 29 | 28 | 1 | 67 |
| Castro (14) | 18 | 6 | 287 | 2 | 313 |
| Li (31) | 88 | 13 | 480 | 48 | 629 |
| Su (24) | 29 | 2 | 124 | 0 | 155 |
| Li (9) | 21 | 17 | 62 | 0 | 100 |
| Hong (13) | 21 | 6 | 214 | 3 | 244 |
| Zhu (23) | 167 | 43 | 855 | 67 | 1132 |
| TRUST | | | | | |
| Zhu (23) | 160 | 50 | 859 | 63 | 1132 |
| Jiang (10) | 18 | 1 | 20 | 0 | 39 |
| Su (7) | 21 | 4 | 95 | 3 | 123 |
| Zheng (18) | 81 | 22 | 151 | 1 | 255 |
| Treponemal Tests | | | | | |
| ELISA | | | | | |
| Lee (19) | 6 | 9 | 513 | 11 | 539 |
| Su (24) | 30 | 1 | 48 | 76 | 155 |
| Li (9) | 36 | 2 | 44 | 18 | 100 |
| Su (7) | 25 | 0 | 43 | 55 | 123 |
| EIA | | | | | |
| Guarner (17) | 23 | 1 | 36 | 0 | 60 |
| Lin (22) | 17 | 0 | 13 | 15 | 45 |
| INNO-LIA | | | | | |
| Guarner (17) | 12 | 1 | 18 | 0 | 31 |
| Dumaresq (27) | 20 | 0 | 8 | 55 | 83 |
| FTA-ABS | | | | | |
| Lin (22) | 57 | 4 | 244 | 51 | 356 |
| Gonzalez (33) | 32 | 0 | 10 | 8 | 50 |
| Dumaresq (27) | 23 | 0 | 9 | 68 | 100 |
| Lee (19) | 14 | 1 | 522 | 2 | 539 |
| Merins (11) | 34 | 4 | 6 | 23 | 67 |
| Lee (13) | 23 | 1 |  |  | 24 |
| Castro (14) | 16 | 0 |  |  | 16 |
| Woehrl (15) | 42 | 1 |  |  | 43 |
| Li (31) | 96 | 5 | 208 | 320 | 629 |
| MHA-TP | | | | | |
| Lee (19) | 11 | 4 | 521 | 3 | 539 |
| Castro (4) | 15 | 1 |  |  | 16 |
| TPPA | | | | | |
| Marra (32) | 17 | 91 | 787 | 39 | 934 |
| Guarner (17) | 10 | 2 | 18 | 0 | 30 |
| Lin (22) | 56 | 5 | 244 | 51 | 356 |
| Zhang (25) | 134 | 15 |  |  | 149 |
| Lin (16) | 29 | 14 | 43 | 0 | 86 |
| Dumaresq (27) | 15 | 7 | 38 | 40 | 100 |
| Lu (26) | 45 | 5 | 42 | 8 | 100 |
| Castro (4) | 16 | 0 |  |  | 16 |
| Jiang (10) | 33 | 0 | 20 | 14 | 67 |
| Li (31) | 99 | 2 | 222 | 306 | 629 |
| Li (9) | 38 | 0 | 53 | 9 | 100 |
| Zheng (18) | 103 | 0 | 152 | 0 | 255 |
| TPHA | | | | | |
| Salamano (30) | 7 | 7 |  |  | 14 |
| Woehrl (15) | 42 | 1 |  |  | 43 |
