# Supplementary material for: Performance of the nontreponemal tests and treponemal tests on cerebrospinal fluid for the diagnosis of neurosyphilis: A meta-analysis
Source: Front Public Health. 2023 Feb 2;11:1105847. doi: 10.3389/fpubh.2023.1105847 (PMC9932918; doi:10.3389/fpubh.2023.1105847)
Supplement: Supplementary Table S3 — Quality of studies. [file Table_3.DOCX]

**Table S3**

Quality of studies

| author | Risk of bias | | | | Applicability concerns | | |  |
| --- | --- | --- | --- | --- | --- | --- | --- | --- |
|  | Patient selection | Index test | Reference standard | Flow and timing | Patient selection | Index test | Reference standard |  |
| Salle (3) | U | L | L | L | L | L | L |  |
| Marra (32) | L | L | L | U | L | L | L |  |
| Gonzalez (33) | H | H | L | L | L | U | L |  |
| Li (31) | L | L | L | L | L | L | L |  |
| Lu (26) | H | H | L | L | U | L | L |  |
| Su (24) | L | U | L | L | L | L | L |  |
| Guarner (18) | H | L | L | U | L | L | L |  |
| Vanhaecke (28) | H | U | L | L | U | L | L |  |
| Lin (17) | U | L | L | L | L | L | L |  |
| Wang (21) | U | U | L | L | L | L | L |  |
| Salamano (30) | L | U | L | L | L | L | L |  |
| Merins (12) | L | L | L | L | L | L | L |  |
| Li (10) | U | L | L | L | L | L | L |  |
| Chan (9) | L | L | L | L | L | L | L |  |
| Zhu (4) | L | L | L | U | L | L | L |  |
| Su (8) | L | L | L | L | L | L | L |  |
| Zhang (25) | L | L | L | L | L | L | L |  |
| Dumaresq (27) | L | L | L | L | L | L | L |  |
| Lin (23) | L | L | L | L | L | L | L |  |
| Jiang (11) | U | U | L | L | L | L | L |  |
| Marra (22) | U | L | L | L | L | L | L |  |
| Zheng (19) | H | L | L | L | H | L | L |  |
| Hong (14) | H | L | L | L | L | L | L |  |
| Castro (15) | U | L | L | L | L | L | L |  |
| Paraskevas (29) | U | U | L | L | L | U | L |  |
| Castro (7) | H | L | L | L | L | L | L |  |
| Woehrl (16) | L | L | L | L | L | L | L |  |
| Lee (20) | U | L | L | L | L | L | L |  |
| Lee (13) | L | H | L | L | L | U | L |  |
| H = high risk of bias; L = low risk of bias; U = unclear risk of bias. | | | | | | | | |
